# Influence of Carbohydrates on Secondary Metabolism in *Fusarium avenaceum*

**DOI:** 10.3390/toxins5091655

**Published:** 2013-09-23

**Authors:** Jens Laurids Sørensen, Henriette Giese

**Affiliations:** Department of Biotechnology, Chemistry and Environmental Engineering, Aalborg University, Sohngaardsholmsvej 49, Aalborg DK-9000, Denmark; E-Mail: hgiese@bio.aau.dk

**Keywords:** secondary metabolites, moniliformin, enniatin, carbon, regulation, activation, *creA*

## Abstract

*Fusarium avenaceum* is a widespread pathogen of important crops in the temperate climate zones that can produce many bioactive secondary metabolites, including moniliformin, fusarin C, antibiotic Y, 2-amino-14,16-dimethyloctadecan-3-ol (2-AOD-3-ol), chlamydosporol, aurofusarin and enniatins. Here, we examine the production of these secondary metabolites in response to cultivation on different carbon sources in order to gain insight into the regulation and production of secondary metabolites in *F. avenaceum*. Seven monosaccharides (arabinose, xylose, fructose, sorbose, galactose, mannose, glucose), five disaccharides (cellobiose, lactose, maltose, sucrose and trehalose) and three polysaccharides (dextrin, inulin and xylan) were used as substrates. Three *F. avenaceum* strains were used in the experiments. These were all able to grow and produce aurofusarin on the tested carbon sources. Moniliformin and enniatins were produced on all carbon types, except on lactose, which suggest a common conserved regulation mechanism. Differences in the strains was observed for production of fusarin C, 2-AOD-3-ol, chlamydosporol and antibiotic Y, which suggests that carbon source plays a role in the regulation of their biosynthesis.

## 1. Introduction

*Fusarium avenaceum* is among the most frequent fungal species encountered on small grain cereals and maize in temperate climate zones such as Northern Europe and Canada [1,2,3,4,5]. The species are pathogenic to diverse plants ranging from apples [6], rutabaga [7], potato tubers [8] and mung beans [9]. *F. avenaceum* is able to produce several bioactive secondary metabolites including moniliformin, fusarin C, antibiotic Y, 2-amino-14,16-dimethyloctadecan-3-ol (2-AOD-3-ol), chlamydosporol, aurofusarin and enniatins [6,10].

**Figure 1:**
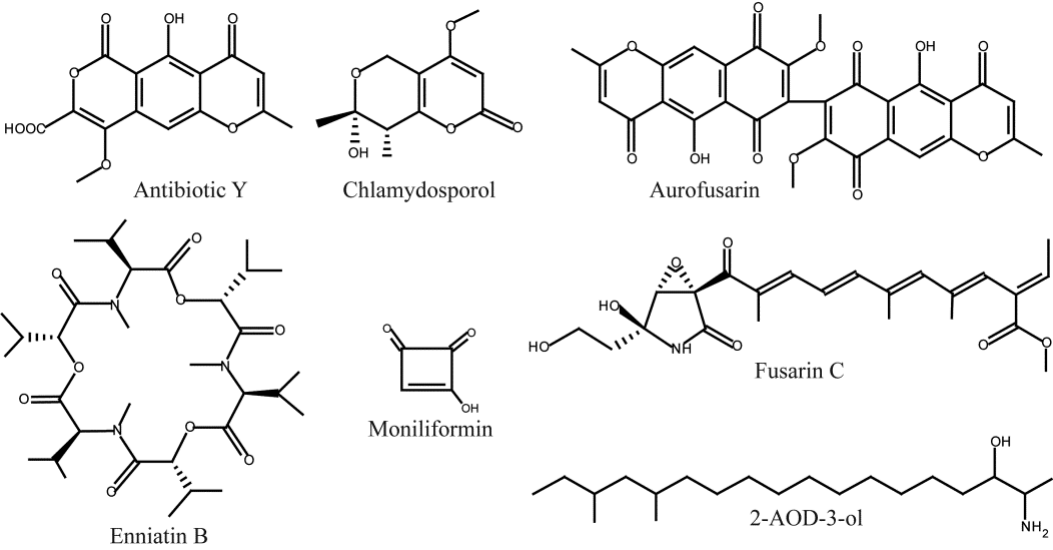
Structures of secondary metabolites produced by *F. avenaceum*.

Several of these attract attention due to their presence in food and feed sources and their potential harmful effects on human and animal health. Moniliformin and enniatins have been characterized as emerging mycotoxins [11,12]. Some of the compounds co-occur in infected plants [6,13], but little is known about the regulatory patterns which control production of secondary metabolites in *F. avenaceum*. Production of secondary metabolites is influenced by global regulators which directly or indirectly activate the respective genes or gene clusters [14]. The global regulators respond to a variety of abiotic components including pH, light, temperature, water activity and nutrient availability [15]. Carbon and nitrogen sources are known to influence production of the trichothecene deoxynivalenol and the polyketide fusarielin H in *F. graminearum* [16,17,18]. AreA, the global nitrogen regulator has been shown to affect trichothecene production in *F. graminearum* [19] and bikaverin and gibberellins in *F. fujikuroi* [20,21]. A link between AreA and the global carbon catabolite repressor, CreA, has been found in *Aspergillus nidulans* with regard to arginine catabolism [22] and lactose has been used as a substrate for penicillin production in *Penicillium chrysogenum* [23]. There is little information on how CreA influence secondary metabolite production in fungi. In *F. avenaceum* water activity and temperature has been shown to have a limited influence on production of antibiotic Y, moniliformin and enniatins [24]. As *F. avenaceum* is able to colonize a diverse range of plants and tissues with different carbon composition we want to gain information on how production of known secondary metabolites in this fungal species is influenced by carbon source. 

## 2. Results and Discussion

### 2.1. Quantification of Secondary Metabolites

**Figure 2:**
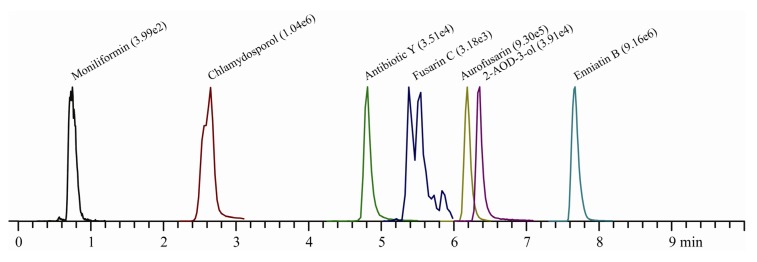
LC-MS/MS detection of quantification traces for moniliformin, fusarin C, antibiotic Y, (2-AOD-3-ol), chlamydosporol, aurofusarin and enniatin B (ion count) produced by *F. avenaceum* (IBT 40847) grown on mannose.

A LC-MS/MS method was developed for quantification of moniliformin, fusarin C, antibiotic Y, 2-AOD-3-ol, chlamydosporol, aurofusarin and enniatins. In order to obtain the optimal quantification conditions the MS/MS parameters and settings were automatically adjusted for each compound (Table 1 and Figure 2). 

**Table 1:** Parameters for selected reaction monitoring (SRM) of *F. avenaceum* secondary metabolites.

Compound	RT (min)^a^	Precursor ion (m/z)	Product ions (m/z)^b^	S-lens (eV)	CID (eV)^c^
Moniliformin	0.75	97.0 [M − H]^−^	41.1	38	18
Chlamydosporol	2.65	227.1 [M + H]^+^	167.7/77.1	63	36/15
Antibiotic Y	4.81	319.1 [M + H]^+^	287.1/175.1	79	22/30
Fusarin C	5.38–5.54	432.2 [M + H]^+^	185.1/213.2	64	29/25
Aurofusarin	6.19	571.1 [M + H]^+^	556.2/485.2	200	34/24
2-AOD-3-ol	6.36	314.4 [M + H]^+^	69.1/97.1	76	20/27
Enniatin B	7.67	657.5 [M + NH_4_]^+^	196.2/214.2	136	30/31
Enniatin B1	7.87	671.5 [M + NH_4_]^+^	196.2/214.2	110	30/24
Enniatin A1	8.10	685.5 [M + NH_4_]^+^	210.1/228.2	113	30/30
Enniatin A	8.21	699.5 [M + NH_4_]^+^	210.1/228.2	115	24/25
Notes: ^a^: Retention time; ^b^: Quantifier/qualifier ions; ^c^: Collision induced dissociation energy for quantifier/qualifier ions.					

Although aurofusarin, chlamydosporol and 2-AOD-3-ol detections were adjusted based on a fungal extract we were able to quantify these secondary metabolites specifically without interference from other contaminating compounds. With the fusarin C settings we detected 2–3 peaks in all *F. avenaceum* extracts suggesting the presence of compounds with similar mass and fragmentation pattern such as the (7*Z*)- and (5*Z*)-fusarin C isoforms. This has previously been observed by a LC-MS/MS method specifically developed for fusarin C [25]. Two overlapping peaks were detected for chlamydosporol probably originating from the minor and major chlamydosporol epimers [26].

### 2.2. Growth and Production of Secondary Metabolites

Ergosterol levels were measured to quantify fungal growth of the three *F. avenaceum* strains on seven monosaccharides (arabinose, xylose, fructose, sorbose, galactose, mannose and glucose), five disaccharides (cellobiose, lactose, maltose, sucrose and trehalose) and three polysaccharides (dextrin, inulin and xylan). Glucose and fructose are predicted to act as repressors of *creA* and arabinose as a derepressor [27]. Lactose is reported to be a poor carbon source for fungal growth and to act as a derepressor of *creA* [28]. The three strains were able to utilize all carbon sources and growth was recorded visually and by ergosterol determination of all samples. The Ergosterol measurements showed that *F. avenaceum* strains IBT 8500 and IBT 40847 had similar growth patterns and generally grew better on the three polysaccharides than the mono- and disaccharides. Conversely strain IBT 5001 had slightly higher ergosterol levels when grown on mono- and disaccharides.

**Figure 3:**
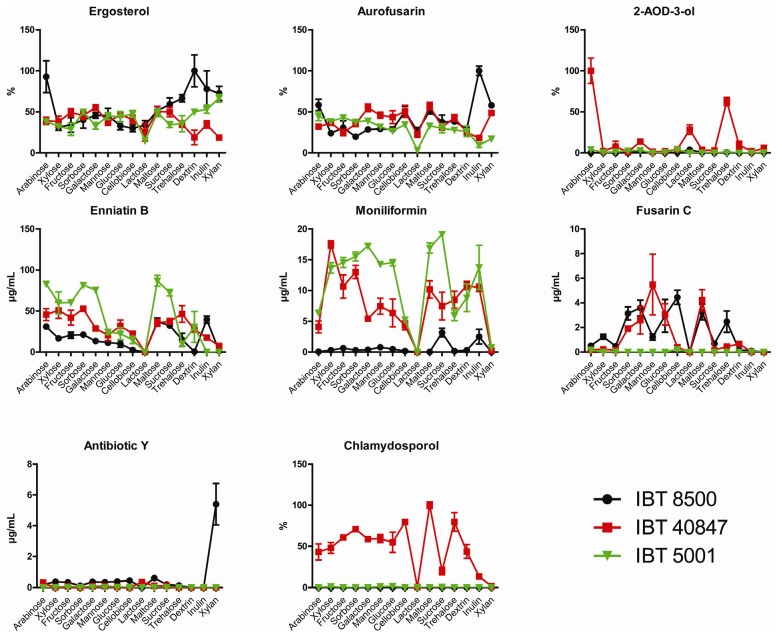
Production of secondary metabolites of the three *F. avenaceum* strains growing on different carbon sources. Error bars indicate standard error of mean of five replicates. Ergosterol, aurofusarin, 2-AOD-3-ol and chlamydosporols were quantified relatively and the maximum mean was set to 100%.

The developed LC-MS/MS method was used to measure production of secondary metabolites by three *F. avenaceum* strains in response to cultivation on the different carbon sources. The three strains exhibited some variations in preferred carbon source for production of the secondary metabolites. Aurofusarin was produced on all carbon sources by the three strains, where the highest levels were observed on inulin for IBT 8500, maltose for IBT 40847 and arabinose for IBT 5001. Lactose was, however, the poorest carbon source for aurofusarin production for all three strains. Strains IBT8500 and IBT 5001 only produced trace amounts of 2-AOD-3-olcompared to IBT 40847. Production of 2-AOD-3-ol in IBT 40847 seemed to be dependent on carbon source with mannose as the best substrate. Enniatin were produced in successively decreasing amounts in the sequence B > B1 > A1 > A, as observed previously [6], and therefore only enniatin B levels are shown. Production of enniatin B was similar in IBT 8500 and IBT 40847 where maltose, sucrose and inulin resulted in high levels. Enniatin B was produced in high levels on maltose and sucrose as well as on trehalose, xylose and sorbose by strain IBT 40847. Lactose was a poor substrate for enniatin B and moniliformin production in all strains. The best carbon source for moniliformin production was sucrose in IBT 8500 and IBT 5001, whereas IBT 40847 had highest production when grown on xylose. Cellobiose resulted in the highest levels of fusarin C in IBT 8500 and 5001, while it was mannose for IBT 40847. Antibiotic Y was not produced by IBT 5001 and only sparsely by IB T 40847 (max 0.35 µg/mL) under the tested nutrient conditions and lactose and arabinose appeared to be the best carbon source. Production of antibiotic Y was highly induced on xylan in IBT 8500 compared to the other carbon sources. Here, only low levels were observed as was the case for the two other strains under all the tested conditions. IBT 8500 did not produce chlamydosporol in these experiments and this compound was only detected in small amounts in IBT 5001 on four carbon sources (xylan, mannose, glucose and sucrose). IBT 40847 was a strong producer of chlamydosporol and produced it on all carbon sources, except lactose. The production of enniatin B and moniliformin followed a similar pattern in all three strains, which could suggest that they are regulated by the same mechanisms through a conserved pathway in *F. avenaceum* that is independent on *creA*. This means that moniliformin and enniatin are likely to co-occur in plants infected with *F. avenaceum*. In contrast to this the strains differed in the production of fusarin C, antibiotic Y and chlamydosporol suggesting that regulation has evolved separately in the three strains. Variation between strains of the same species in production of secondary metabolites in response to different carbon sources was also observed for DON production in *F. graminearum* [17], which illustrates the need to include several strains when examining the effects of nutrients on secondary metabolism in a species. 

Lactose provided the poorest source of carbon for production of secondary metabolites in the tested strains as aurofusarin, chlamydosporol, enniatin B, moniliformin and fusarin C were all produced in low amounts. Lactose has previously been shown to stimulate production of lovastatin in *Aspergillus terreus* [29] and have been used as substrate for production of penicillin [23]. The negative effect of lactose on secondary metabolism in *F. avenaceum* observed in the present study is therefore not common feature for all fungi. Lactose is not a natural substrate for plant pathogenic fungi like *F. avenaceum* and the utilization of this carbon source may differ from fungi that are adapted to this substrate. However, little information is currently available on how individual mono-, di- and polysaccharides affect secondary metabolism and which mechanisms are involved. Carbon clearly has an effect on secondary metabolism in filamentous fungi but the regulatory pathways are probably interlinked with global regulators. 

## 3. Experimental Section

### 3.1. Chemicals

All chemical solvents were obtained from Thermo Fisher Scientific (San José, CA, USA) and carbon sources were purchased from Sigma-Aldrich (St. Louis, MO, USA) and Merck (Whitehouse Station, NJ, USA). Reference standards of enniatin mix (17% A, 34 % A1, 24% B and 26% B1) and antibiotic Y were purchased from Bioaustralis (Smithfield, Australia), moniliformin from Sigma-Aldrich, whereas fusarin C was available from previous studies [30].

### 3.2. Fungal Isolates and Cultivation

Three *F. avenaceum* strains (IBT 8500, 40847 and 5001) were selected from the IBT collection at the Technical University of Denmark. Conidia were produced by growing the strains in a 250 mL baffled flasks containing 50 mL liquid sporulation medium [31] for three days at 20 °C in the dark at 150 rpm. The spores were isolated by centrifugation and dissolved in sterile H_2_O to give a final concentration of 1 × 10^6^ spores per mL. Cultivation on different carbon sources was performed as previously described [18]. In brief, twenty μL spore suspension was inoculated to 14 mL culture tubes containing 1 mL modified czapek dox (CZ) medium (pH 6) containing: 30 mg carbon source (l-arabinose, d-xylose, d-fructose, l-sorbose, d-galactose, d-mannose, d-glucose, d-cellobiose, β-lactose, maltose, sucrose, dextrin from corn, d-xylan and d-trehalose); 1.5 mg arginine; 1 mg K_2_HPO_4_; 0.5 mg KCl; 0.5 mg MgSO_4_·7H_2_O; 0.01 mg FeSO_4_·7H_2_O and 1 μL trace solution (1 g ZnSO_4_·7H_2_O and 0.5 g CuSO_4_·5H_2_O in 100 mL H_2_O) and cultivated for 14 days at 25 °C in the dark. 

### 3.3. Chemical Analyses

The cultures were extracted with 3 mL acetonitrile/water/acetic acid (79/20/1) using a Vibracell VC130 sonicator (Sonics & Materials, Inc., Newtown, CT, USA) with an amplitude of 100 for 10 seconds per sample subsequently by rotation at 180 rpm for 1.5 hours. The extracts were spun for two minutes at 12,000 rpm in 2 mL tubes and then transferred to 2 mL HPLC vials for analysis. The samples were analyzed on a dionex UltiMate 3000 UHPLC system (Dionex, Idstein, Germany) connected to a Thermo Vantage triple stage quadrupole mass spectrometer (Thermo Fisher Scientific, San José, CA, USA) with a heated electrospray ionization probe. 5 μL were injected and separated on a Gemini C6-Phenyl 3 μm 2-mm i.d. × 50-mm column (Phenomenex, Torrance, CA, USA) using a constant flow of a 0.4 mL/min and gradient system consisting of A (H_2_O:acetic acid; 99:1) and B (MeCN:H_2_O:acetic acid; 89:10:1), both buffered with 5 mM ammonium acetate. The gradient started at 0% B increasing to 100% over 10 min, which was maintained for three minutes before reverting to 0% B in one minute and recalibrated for two minutes. The following ion source parameters were used for detection: spray voltage (4.5 kV), vaporizer temperature (350 °C), nitrogen sheath gas pressure (30 arbitrary units), nitrogen auxiliary gas pressure (10 arbitrary units), capilliary temperature (270 °C). Argon was used as the collision gas and set to 1.5 mTorr. Collision energy and selected reaction monitoring (SRM) transitions were automatically optimized for moniliformin, fusarin C, antibiotic Y and enniatin A, A1, B and B1, whereas aurofusarin, chlamydosporol and 2-AOD-3-ol was tuned from extract of *F. avenaceum* grown on yeast extract sucrose agar (Table 1). Ergosterol was measured at 280 nm on an Agilent 1200 LC system (Agilent Technologies, Waldbronn, Germany) equipped with a diode array detector. Five μL extract was injected and separated on a 100 × 2.1 mm kinetex 2.6 μm phenyl-hexyl (Phenomenex, Torrance, CA, USA) using a flow of 0.4 mL/min with a linear water-acetonitrile gradient, where both eluents were buffered with 50 ppb trifluoro acetic acid. The gradient started at 5% acetonitrile and reached 100% in 10 min, which was held for 2 min.

## 4. Conclusions

The results showed that *F. avenaceum* strains IBT 8500 and IBT 40847 grew better on the three polysaccharides than the mono- and disaccharides, whereas strain IBT 5001 had slightly higher ergosterol levels when grown on mono- and disaccharides.

All three strains were able to grow and produce aurofusarin on all the tested carbon sources. Lactose resulted in the lowest production of aurofusarin, chlamydosporol, enniatin B, moniliformin and fusarin C levels under the tested conditions, which can be due to reduced growth or direct or indirect effects on biosynthesis of secondary metabolites in *F. avenaceum*. Enniatin B and moniliformin were produced on most of the carbon sources although variation between the strains was observed. Antibiotic Y, 2-AOD-3-ol and chlamydosporol levels varied more between the different carbon sources in each strain, which could suggest that production of these secondary metabolites is under the influence of regulators which respond to carbon source.
